# Rapid Assessment of Cerambycid Beetle Biodiversity in a Tropical Rainforest in Yunnan Province, China, Using a Multicomponent Pheromone Lure

**DOI:** 10.3390/insects12040277

**Published:** 2021-03-24

**Authors:** Jacob D. Wickham, Rhett D. Harrison, Wen Lu, Yi Chen, Lawrence M. Hanks, Jocelyn G. Millar

**Affiliations:** 1 Key Laboratory of Analytical Chemistry for Living Biosystems, Institute of Chemistry, Chinese Academy of Sciences, Beijing 100190, China; chenyi@iccas.ac.cn; 2 Kunming Institute of Botany, Chinese Academy of Sciences, Heilongtan, Kunming 650201, Yunnan, China; r.harrison@cgiar.org; 3 Department of Plant Protection, College of Agriculture, Guangxi University, Nanning 530005, Guangxi, China; luwenlwen@163.com; 4 Department of Entomology, University of Illinois at Urbana-Champaign, Urbana, IL 61801, USA; hanks@illinois.edu; 5 Department of Entomology, University of California, Riverside, CA 92521, USA

**Keywords:** Cerambycidae, generic pheromone lures, rapid biodiversity assessment, enantiomeric synergism, *Perissus mimicus*, *Rhaphuma horsfieldi*

## Abstract

**Simple Summary:**

The Cerambycidae comprise a diverse (>35,000 species) family of wood-boring beetles. Many are of concern as invasive species because their long-lived larvae are readily transported around the world concealed in wooden products and packing materials. Over the past two decades, our understanding of cerambycid pheromone chemistry has advanced rapidly, with pheromone structures now described for several hundred species. Furthermore, mixtures of cerambycid pheromones have been shown to potentially act as effective multispecies lures. In this study, traps baited with generic lures deployed at ground level and in the tree canopy in 22 randomly located permanent plots in a nature reserve in Yunnan, China, captured 4541 beetles of 71 species. Using Hierarchical Modeling of Species Communities, we developed informative models for 18 species and demonstrated that trap height, slope, elevation, and leaf-area index were important determinants of cerambycid beetle distribution. Our results demonstrate the potential for using generic lures to detect and monitor cerambycid populations at ports of entry, and for the study of cerambycid beetle ecology.

**Abstract:**

The Cerambycidae comprise a large and ecologically important family of wood-boring beetles. The purpose of this study was to examine the effectiveness of a generic lure as a potential monitoring tool. Working in a subtropical forest in southwest China, we set traps baited with generic lures at ground level (1 m) and canopy height (~18 m) across 22 randomly located forest plots (12 regenerating forest, 10 mature forest). Three stations were established per plot and each plot was trapped for 7 days in May–June 2013. In total, 4541 beetles of 71 species were caught, including 26 species with 10 or more individuals. We used Hierarchical Modeling of Species Communities (HMSC) to analyze the data and produced informative models for 18 species, showing that trap height, slope, elevation, and leaf-area index were important determinants of cerambycid distribution. Our results demonstrate the potential for using generic lures to detect and monitor cerambycid populations, both for regulatory purposes and for the study of cerambycid beetle ecology. Further research should focus on refining lure blends, and on repeated sampling to determine temporal and spatial dynamics of cerambycid communities.

## 1. Introduction

The Cerambycidae, commonly known as longhorned beetles, comprise a large family of woodborers with >35,000 described species in eight subfamilies [1]. The endophytic larvae develop in woody plants [2], and while some species have become globally important as invasive forest pests [3], they generally perform valuable ecosystem services by initiating the degradation of woody plants and can be indicators of forest ecosystem health [4]. The larvae can be long-lived and, when concealed in wooden dunnage and other packing materials, are readily transported in global commerce [2]. Consequently, cerambycid beetles are among the most frequently intercepted phytophagous species at international ports of entry, with numerous cases of accidental introductions resulting in invasive outbreaks. Thus, the development of surveillance tools based on semiochemically-baited traps for detection and prevention of introductions is of high importance. Conversely, such tools may also be useful in ecological studies, and for monitoring endangered species [5].

Less than two decades ago, pheromones had been reported for only nine species of cerambycids [6]. Since then, there has been rapid progress in our understanding of cerambycid semiochemistry, with the identification of pheromone-based attractants for several hundred species [7,8], and these compounds are increasingly being incorporated into quarantine surveillance and monitoring programs [9,10]. The cumulative body of research suggests that pheromone structures are often conserved within cerambycid genera and tribes, with related species often using similar or the same chemical structures in their pheromone blends. For example, many species in the subfamily Cerambycinae utilize short-chain (6–10 carbon) hydroxyketones and diols as pheromone components, whereas terpenoids such as fuscumol [(*E*)-6,10-dimethyl-5,9-undecadien-2-ol] and fuscumol acetate are recurring motifs in the pheromones of species in the subfamilies Spondylidinae and Lamiinae [7,8]. Within the Lamiinae, hydroxyethers such as monochamol [2-(undecyloxy)ethanol] also appear to be shared among a number of species [7,8].

As a result of this evolutionary conservatism in pheromone chemistry among cerambycid species, traps baited with common pheromone compounds such as those described above frequently catch several species simultaneously e.g., [11,12,13,14]. For example, in a field trial conducted in Yunnan Province, China, using traps baited with ten test components, a total of 1526 cerambycids representing 71 species were captured at a single site in the Bulong Nature Reserve [13]. Of these, fourteen species were significantly attracted to at least one of the test compounds, accounting for 92% of the specimens collected [13]. In particular, compounds with a 3-hydroxyalkan-2-one or 2,3-alkanediol motif attracted significant numbers of both sexes of eight species in the subfamily Cerambycinae, including species in the genera *Demonax*, *Rhaphuma*, and *Xylotrechus* [13]. One species, *Rhaphuma horsfieldi* (White), was strongly attracted to two homologous test compounds (racemic *syn*-2,3-hexanediol and *syn*-2,3-octanediol) [13]. Within the Lamiinae, males and females of five species, including *Acalolepta formosana* (Breuning), *Monochamus bimaculatus* Gahan, *Pharsalia subgemmata* (Thomson), *Pseudomacrochenus antennatus* (Gahan), and *Xenohammus bimaculatus* Schwarzer, were significantly attracted to monochamol, suggesting the potential of this chemical as a general attractant rather than as a *Monochamus*-specific attractant, as its name might suggest [15,16,17,18]. Unexpectedly, males of *Megopis costipennis* White, subfamily Prioninae, were attracted to racemic *anti*-2,3-octanediol, and further field tests in 2013 determined that males were equally attracted to (2*R*,3*S*)-2,3-octanediol and its racemate, but not to the (2*S*,3*R*)-2,3-octanediol enantiomer. Females were found to produce (2*R*,3*S*)-2,3-octanediol as a sex pheromone [19]. Previously, 2,3-octanediols had only been reported as male-produced aggregation pheromone components for cerambycine species, although the homologous 2,3-hexanediols are known female-produced sex pheromones or sex attractants for several species in the prionine genus *Tragosoma* [20]. This suggested that C_6_ and C_8_ diols might have potential for use as generic lures for both cerambycines and prionines. However, perhaps the most promising generic component is 3-hydroxyhexan-2-one, which has been reported as a pheromone component for numerous cerambycine species from six continents [7].

A number of recent studies also have demonstrated that several cerambycid pheromone components can be combined to make “generic” lures which can attract multiple species simultaneously (North America, [11,21,22,23]; Europe, [9]). Thus, a primary objective of the study described herein was to follow up on the results from Wickham et al., 2014, [13], by testing the efficacy of a multi-component blend of pheromone components, selected from the most attractive single components in that study. In particular, we wanted to test such blends as a means to rapidly assess the cerambycid biodiversity in a survey conducted across 22 randomly located, permanent plots in the Bulong Nature Reserve in Yunnan Province, China [24]. The plots included both mature forest with a closed canopy and regenerating forest with an open canopy.

In addition, several recent studies have shown that trap placement can significantly affect trap captures of arboreal insects [25,26], including cerambycids, with some species being caught primarily at ground level, whereas others were caught preferentially by traps in the canopy [10,27,28,29,30]. These reports suggest that different species prefer different strata within the tree canopy, possibly as a function of their differing requirements for either standing or fallen trees as hosts, and trunks versus branches. Thus, as a second objective, we examined the effects of placing pheromone-baited traps at two different heights (~1 and ~18 m) in the forest canopy. Furthermore, variation in other characteristics of the sites (e.g., elevation, slope, and leaf area index) allowed us to conduct more detailed analyses of how the distribution of beetles was affected by environmental characteristics.

In a final experiment following up on previous work [19], we tested the relative attractiveness of racemic *anti*-2,3-octanediol versus each of the two individual enantiomers [(2*R*,3*S*)-2,3-octanediol and (2*S*,3*R*)-2,3-octanediol], targeting cerambycine and prionine species.

## 2. Materials and Methods

### 2.1. Sources of Chemicals

Racemic *syn*- and *anti*-2,3-hexanediols were prepared by OsO_4_-catalyzed oxidation of (*E*)- or (*Z*)-2-hexene respectively, as described in Lacey et al., 2004, [31]. Racemic *syn*-and *anti*-2,3-octanediols were synthesized in analogous fashion from (*E*)- or (*Z*)-2-octene. Racemic 3-hydroxyhexan-2-one and monochamol were purchased from Bedoukian Research (Danbury CT, USA). (2*S*,3*R*)-2,3-Octanediol and (2*R*,3*S*)-2,3-octanediol were synthesized as described in Wickham et al. (2014) [13].

### 2.2. Field Trials

From 17 May–12 June 2013, a six-component lure including 50 mg each of *syn*-2,3-hexanediol, *anti*-2,3-hexanediol, *syn*-2,3-octanediol, *anti*-2,3-octanediol, racemic 3-hydroxyhexan-2-one, and 25 mg monochamol was tested for 7 days per site in 22 randomly selected permanent plots in the Bulong Nature Reserve (21°30′29.39″ N, 100°29′50.60″ E; elevation ~1600 m), at the southern tip of Yunnan Province in China. Ten sites were classified as mature forest with a closed canopy, whereas 12 sites were considered regenerating forest with an open canopy. The reserve (40 km in diameter) is a seasonal tropical montane rain forest, which grades into broadleaf evergreen forest on slopes and ridges, with mixed old-growth and secondary forest interspersed with tea plantations and grasslands [24]. Average summer (May to June) temperatures are 15–21 °C [32]. Lures consisted of six low-density polyethylene press-seal baggies (5 × 7.5 cm, 0.05 mm wall thickness; #01-816-1A, Fisher Scientific, Pittsburgh, PA, USA), each separately containing 1 mL of an ethanol solution of a test compound. Controls consisted of six individual baggies loaded only with 1 mL ethanol.

Each permanent plot was subdivided into a 3 × 3 grid of 9 subplots, spaced 50 m apart (for site characteristics and tree species composition, see Table S1). Fluon-coated black cross-vane panel traps [33] (AlphaScents, Portland, OR, USA; see https://www.alphascents.com/panel-trap-black-complete.html, (accessed on 18 Mar 2021), and Figure S1) were hung from trees at ground level (~1 m) and from tree branches in the canopy (~18 m) on a diagonal within subplots 1, 5, and 9 for a total of six traps per site, with a distance between subplots of >70 m and vertical distance between traps of ~17 m. Trap cups were filled with soapy water to kill and preserve captured beetles. Traps were emptied on day 7, and then moved to a new site. To minimize temporal variation and simulate a rapid assessment, the study was completed within the shortest timeframe possible (27 d) with a crew of three people. The order in which plots were surveyed was randomized, but all subplots within a plot were surveyed at the same time for logistical reasons.

A second experiment consisted of a targeted study for prionine and cerambycine species (see [19]), with the goal of comparing attraction of species to the individual enantiomers (2*S*,3*R*)-2,3-octanediol and (2*R*,3*S*)-2,3-octanediol), the racemic mixture of the two enantiomers (i.e., *anti*-2,3-octanediol), and a solvent control. Cross-vane panel traps were hung from trees at a height of ~1 m and lures consisted of the press-seal baggies described above, loaded with the individual enantiomers (25 mg/lure in 1 mL ethanol) or the racemic diol (50 mg/lure in 1 mL ethanol). Control lures contained ethanol only. Traps were deployed at the single site at Bulong Nature Reserve described in Wickham et al. (2016) [19] from 24–31 May 2013 (*n* = 4 replicates per treatment), with traps checked on 27 May and rotated one position to control for spatial effects. Trap cups filled with soapy water were as described above and beetles were collected on 27 and 31 May.

Beetles were identified to species by Wen Lu using the keys of Gressit, 1951, [34], Gressit et al., 1920, [35], Hua et al., 2009, [36], Nga and Long, 2014, [37], and Nga et al., 2016, [38], and voucher specimens were deposited in the museum collections of Guangxi University, Guangxi, China.

### 2.3. Statistical Analyses

We used basic summary statistics to demonstrate the effectiveness of the generic lures. The purpose of further analyses was to demonstrate that data derived from beetle captures using generic lures could be used in biodiversity surveys to reveal information concerning the underlying environmental and biological determinants of community composition.

Our ability to analyze community datasets has advanced considerably in recent years through the development of hierarchical modeling frameworks that enable linear modeling of multivariate species responses. Here we applied Hierarchical Modeling of Species Communities (HMSC; [39]), as implemented through the *R* package HMSC [40]. We tested the hypotheses that (a) trap height, (b) elevation, (c) slope, and (d) leaf area index (LAI) were important determinants of cerambycid community composition. Plot characteristics were obtained from Paudel et al., 2015, [24]. LAI provides a measure of the quality of the forest, with more mature forest supporting a higher LAI, while elevation and slope are often important environmental gradients. Moreover, at our site, forest composition transitions from montane rainforest in valleys to evergreen broadleaf forest at higher elevations, and plant species composition is an important determinant in the distribution of arthropod biodiversity [41,42]. Captures from subplots within a plot were combined for each height, which reduced the number of zeros and produced better response distributions across species. We also removed species with <10 individuals from the analyses to reduce the dimensionality and because, with such low abundances, it is challenging to detect determinants of species distribution. To account for the nested structure of the dataset (two heights per plot), we assigned random effects at the plot and sample levels. In community datasets, sample level random effect represents not only the variation within species (i.e., residual variation) but also the covariation among species. This covariation may represent species interactions (e.g., competition or facilitation) or could represent the effect of a common response to an unspecified factor. HMSC enables models to be structured in a variety of ways through the use of latent variables. For example, autocorrelation among species due to shared ancestry can be modeled given a phylogeny, or the effects of shared traits among species can be considered. In the absence of more detailed data, we used subfamily as a proxy for traits conserved at this taxonomic level.

Our data were zero-inflated and hence we applied a Hurdle model approach and defined a presence-absence model, which in HMSC is specified with a Probit distribution, and an abundance conditional-on-presence model, which we specified as a Lognormal Poisson [43]. These models are independent, and it is of interest to investigate whether similar factors may determine species incidence, as species abundance conditional-on-presence (hereon referred to as ‘abundance’). For example, a species might be found at a particular elevation, but its abundance at that elevation is determined by forest condition. In the maximal model, we specified the main effects of trap height, elevation, slope, and LAI, and all two-way interactions.

For model diagnostics and displaying results, we followed the guidance provided by the HMSC manual [43]. We conducted a five-fold cross-validation to examine predictive performance. In the Probit model, we examined the Tjur *r^2^* across species and in the Lognormal Poisson the *r^2^* [43]. We simplified the models by removing terms one by one, selecting the term with the least support across all species, starting with the higher order terms and respecting the principle of marginality. All analyses were conducted in *R* version 3.6.3 (R Core Team; [44]).

We tested differences in attraction between racemic *anti*-2,3-octanediol and its enantiomers using non-parametric Friedman’s Q test followed by the REGWQ test to analyze the differences in number of individuals attracted to each lure (PROC FREQ, option CMH, SAS Institute; [45]).

## 3. Results

### 3.1. Field Trials

#### 3.1.1. Generic Lures

In the first, rapid survey experiment in which generic lure mixtures were tested for one week per site at each of 22 different sites (22 sites × 6 traps per site × 7 days = 924 trapping days) within the nature reserve, more than 4500 beetles representing 71 cerambycid species were captured (Table 1). Of these 71 species, one species was caught in numbers >1000, 8 were caught in numbers of 101 to 1000, 17 were caught in numbers of 10 to 100, and 45 were caught in numbers of <10. Twenty-two species were represented by singletons.

Eleven of the 14 species that had been attracted in significant numbers to traps baited with single compounds in our 2010 trial [13] were also captured in good numbers in the present study, including five lamiine species (*A. formosana*, *M. bimaculatus*, *P. subgemmata*, *P. antennatus*, *X. bimaculatus*), five cerambycine species (*Demonax gracilestriatus* Gressitt and Rondon, *Demonax theresae* Pic, *R. horsfieldi*, *Rhaphuma laosica* Gressitt and Rondon, *Xylotrechus incurvatus* [Chevrolat]), and the prionine *M. costipennis*. In contrast, the three remaining species captured in significant numbers in 2010 (the cerambycines *Demonax literatus literatus* Gahan, *Demonax ornatus* Pascoe, and *Xylotrechus atronotatus draconiceps* Gressitt) were not caught in the present study. Seven cerambycine species (*Artimpaza lineata* [Pic], *Demonax occultus* Gressitt and Rondon*, Demonax pseudonotabilis* Gressitt, *Xylotrechus lateralis fracturis* Guo and Chen, *Perissus mimicus* Gressitt and Rondon, *Perissus griseus* Gressitt, and *Rhaphuma anongi* Gressitt and Rondon) were caught in significant numbers in the current study, which were not caught at all (first four species) or were caught in low and nonsignificant numbers (latter three species) in the previous study testing single components [13].

#### 3.1.2. Racemic *Anti*-2,3-octanediol versus Its (2*R,*3*S*)- and (2*S,*3*R*)-Enantiomers

In the targeted experiment comparing attraction to racemic *anti*-2,3-octanediol versus the (2*R*,3*S*)- and (2*S*,3*R*)-enantiomers, two cerambycine species in the tribe Clytini were attracted to baited traps in significant numbers (Table 2). Thus, *P. mimicus* was attracted only to the racemic mixture, and not to either of the enantiomers, indicating synergism between enantiomers. A second species, *R. horsfieldi*, was also caught in relatively low numbers. For the latter species, the (2*S*,3*R*)-enantiomer was significantly more attractive than the controls (which caught no beetles). There was no significant difference in attraction between the racemate or either of the enantiomers, nor were the racemate and the (2*R*,3*S*)-enantiomer significantly different than the controls for this species.

### 3.2. Environmental Determinants of Cerambycid Beetle Trap Captures

The best performing incidence model retained all the main effects (trap height, elevation, slope, and LAI) and the interactions between trap height and elevation, and elevation and LAI. For the abundance model, only the main effects of trap height, elevation, slope, and LAI were retained in the best performing model. The incidence model performed reasonably well (predictive Tjur *r*^2^ > 0.10) in 13 species, while the abundance model performed reasonably well (predictive *r*^2^ > 0.10) in 11 species. However, eight species were not well predicted by either model (Table 3). For a full description of the modeling, model evaluation, and model outputs please refer to the online Supplementary Materials. Results presented here were based on species with a predictive Tjur *r*^2^ > 0.10 or *r^2^* > 0.10 for the incidence and abundance models, respectively.

**Figure 1:**
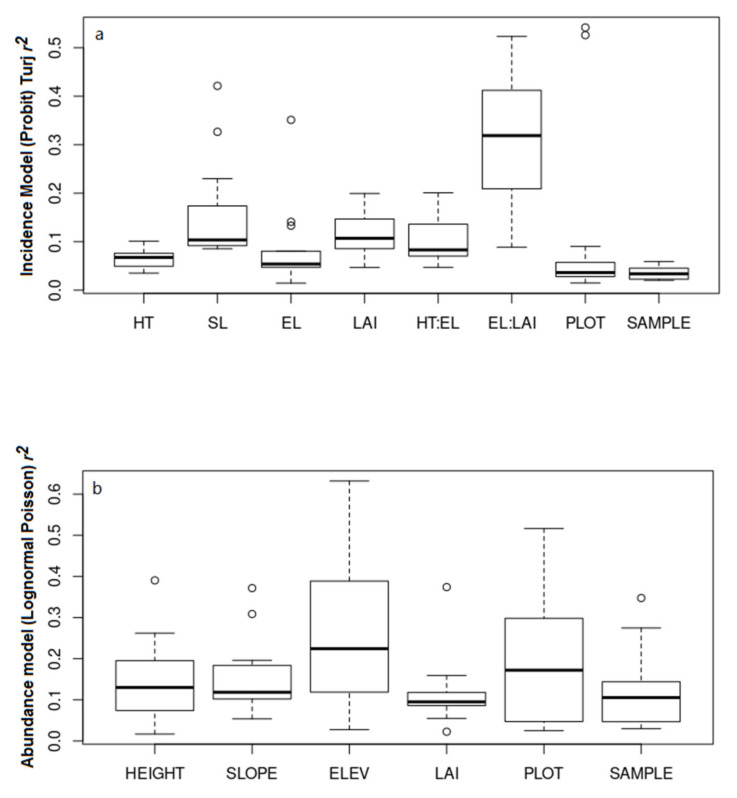
Box and whisker plots illustrating the proportion of variance in trap captures explained by environmental variables across species; (**a**) Incidence model (Probit); (**b**) Abundance model (Lognormal Poisson). Only species with a predictive *r^2^* of >0.10, based on a 5-fold cross-validation, were included. Models were estimated using Hierarchical Modeling of Species Communities (HMSC; Ovaskainen et al. 2017 [39]).

Variance partitioning showed that there was a difference in the importance of factors between the two models (Figure 1). The interaction between Elevation and LAI was by far the most important variable in the incidence model, explaining over 30% of the variance on average across species, while other variables explained around 10% of the variance. The plot level and sample level random variables each explained less than 5% of the variance on average across species. In contrast, in the abundance model, elevation was the most important factor, explaining about 22% of the variance on average across species, while trap height, slope, and LAI explained around half that amount. In the abundance model, the plot level and sample level random variables were also more important, explaining around 15% and 10% of the variance on average across species, respectively.

**Figure 2:**
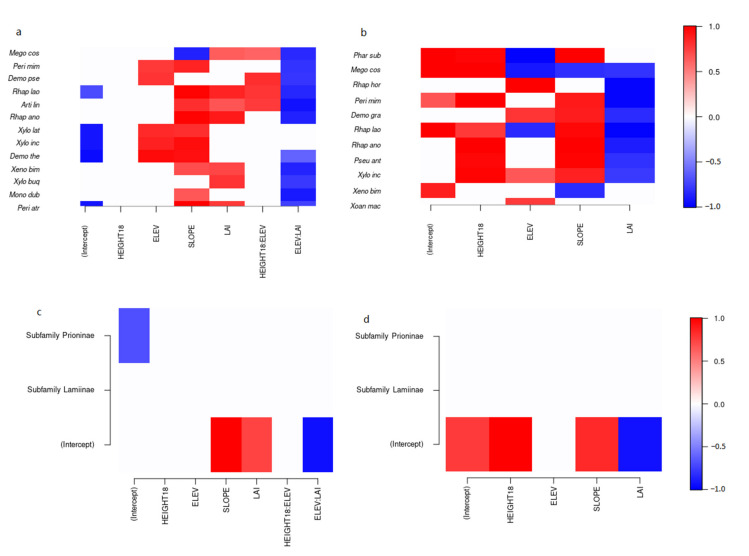
Heatmap illustrating the support (>0.80) for positive or negative *β*-coefficients across species (*y*-axis) and environmental factors (*x*-axis) (**a**,**b**) and for γ-coefficients across cerambycid subfamilies (**c**,**d**) (Cerambycinae was the baseline group and hence represents the intercept, while Prioninae was represented by a single species, *Megopis costipennis*); (**a**,**c**) Incidence model (Probit); (**b**,**d**) Abundance model (Lognormal Poisson). Red indicates a positive coefficient, while blue indicates a negative coefficient. Models were estimated using Hierarchical Modeling of Species Communities (HMSC; [39]). Only those species for which the predictive Tjur *r^2^* or *r^2^*, respectively, based on a 5-fold cross-validation, was >0.10 have been included.

In the incidence model (Figure 2a), there was substantial (>0.80) support for a negative interaction between elevation and LAI for 11 out of 13 species, and support for a positive interaction between trap height and elevation among four species. Seven out of the 13 species showed a positive association with LAI, ten with slope, and five with elevation. One of the 13 species showed strong support for a negative association with slope. Meanwhile, in the abundance model (Figure 2b), there was significant support for a negative association with LAI in eight out of the 11 species, and strong support for positive association with slope in seven species, with elevation in four species, and with trap height in seven species. Two species showed strong support for a negative association with slope and three species with elevation.

Referring to the analysis of gamma coefficients, in the incidence model Cerambycinae showed strong support for a positive association with slope and LAI, and a negative association with the interaction between elevation and LAI (Figure 2c). In the abundance model, Cerambycinae showed strong support for a positive association with trap height and slope, and a negative association with LAI (Figure 2d). There were no strong associations with environmental factors in either model for Lamiinae. The subfamily effect could not be assessed for Prioninae because only one species was caught. In terms of species-species associations (not shown), there were no associations with significant support in the incidence model and only one (between *M. costipennis* and *R. horsfieldi*) in the abundance model.

**Figure 3:**
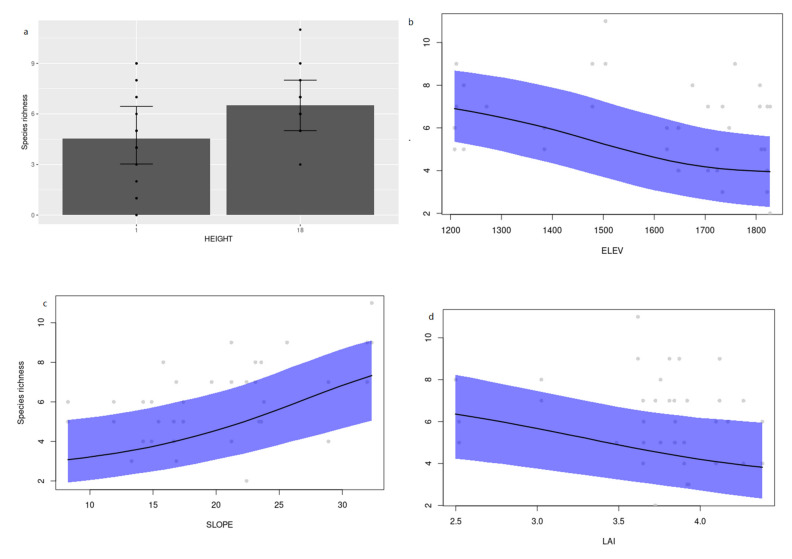
Community responses: Species richness vs. (**a**) Trap height; (**b**) elevation; (**c**) slope; (**d**) leaf area index.

We examined community-level responses to the environmental factors using the models generated. Species richness (incidence model) was higher in canopy traps (Figure 3a) and showed a negative correlation with elevation (Figure 3b) and LAI (Figure 3d), but a positive correlation with slope (Figure 3c). There were no significant community-level associations (total abundance) for the abundance model (not shown).

## 4. Discussion

The results described here suggest that traps baited with multicomponent lures can be a valuable tool for rapidly sampling a representative subset of the cerambycid fauna of a region, at least for the species that respond to pheromones, coupled with random, passive trap captures of species flying at the sampling time. Of the 71 species caught in total, 26 were caught in numbers greater than 10 individuals, with the largest catch being 1601 specimens of *P. subgemmata*. However, as is typical of arthropod surveys in the tropics e.g., [41] a large proportion of species were represented by only a few specimens. An additional 45 species were caught in numbers < 10, and of those, 22 species were represented by a single individual. Overall, this is not surprising given that to date, pheromones or likely pheromones have been identified for only a few hundred of the >35,000 described cerambycid species worldwide [1]. Thus, numerous additional pheromone structures undoubtedly remain to be discovered.

The mix of species caught was similar but not identical to that caught in our previous study ([13], see Table S2) in which lures consisted of the individual components of the blend tested in this study. Here, we trapped only 11 of the 14 species caught in significant numbers in the previous study, which may have two possible explanations. First, it is possible that one or more of the blend components inhibited attraction of the other three species, a phenomenon which has been noted in other studies in which blends of pheromones were tested where cross-attraction is averted by minor components e.g., [46,47,48,49]. Conversely, in the current study, four species were caught in good numbers which had not been caught at all in the 2010 study, potentially suggesting that the pheromones of these species may consist of two or more components of the tested blend that act synergistically, with minimal attraction to the individual components. This synergism of multiple pheromone components has been observed with cerambycid species in several trapping studies [50,51,52,53]. A second factor that may have contributed to the difference in the mix of species caught between the two studies was the timing of deployment of the experiments. That is, the 2010 study was run from 28 May–25 June, whereas the current study was run from 17 May–12 June, i.e., approximately two weeks earlier. Given that the flight periods of cerambycid species may be only a few weeks long e.g., [54], this difference in the times of deployment may have influenced the species composition seen in each study. Specificity in flight times may also explain the large proportion of species caught in low numbers if trapping periods only overlapped with the very beginning or end of the flight periods of these species. Regular monthly monitoring with generic lures, utilizing different colored traps to capture flower-visiting lepturine cerambycids [49,55] or different canopy traps/baits [56,57,58], as well as testing different combinations of components in the lures, would enable researchers to differentiate between these possibilities and enhance understanding of cerambycid ecology in the nature reserve. Specifically, utilizing different colored traps would potentially include species in the flower-visiting subfamily Lepturinae, that were not targeted with the current trapping design.

Our results also highlight the value of studies such as this in providing leads as to the possible pheromone structures of new species. In particular, of the 26 species caught in numbers > 10 individuals, pheromones have been formally identified for only two species, *M. costipennis* ((2*R*,3*S*)-2,3-octanediol; [19]), and *Xylotrechus chinensis* (Chevrolat) ((*syn*-2,3-octanediol, [*S*]-2-hydroxyoctan-3-one, and 3-hydroxyoctan-2-one; [59,60]). Eleven additional species have been attracted in significant numbers to traps baited with known cerambycid pheromones in previous studies (species in bold font in Table 1). However, for the remaining 13 species caught in numbers >10 individuals, the data presented here represent the first possible leads to their pheromone structures. For at least some of these species, which were caught in numbers > 100 (*A. lineata*, *D. occultus*, *P. mimicus*, *R. anongi, R. horsfieldi*, *R. laosica*), it is very likely that the major component(s) of their pheromones were present in the blend that was tested. Leads on the identification of new pheromone structures are particularly important in the context of developing effective lures for known invasive species, such as Asian species in the genera *Chlorophorus*, *Xylotrechus*, and *Acalolepta* e.g., [61,62,63].

In the field trial testing racemic *anti*-2,3-octanediol versus the individual (2*R*,3*S*)- and (2*S*,3*R*)-enantiomers, in addition to trapping a large number of the prionine *M. costipennis* (as previously reported by [19]), we also caught significant numbers of the cerambycines *P. mimicus* and *R. horsfieldi*, both in the tribe Clytini. This was remarkable for two reasons. First, it is extremely unusual for the same compound to be used as a female-produced sex pheromone by species in one subfamily, and as a male-produced aggregation-sex pheromone by species in another subfamily. However, there is a precedent for this phenomenon within the Cerambycidae, whereby 2,3-hexanediols are used as sex pheromones by prionine species in the genus *Tragosoma* [20] and as aggregation-sex pheromones by a number of cerambycine species (summarized in [7]). Second, instances of synergism between enantiomers are also rare in insect pheromones, but several recent examples have been identified in the Cerambycidae. For example, Meier et al. 2016, 2020, reported several cases of the enantiomers of fuscumol or fuscumol acetate acting synergistically as pheromone components in several lamiine species [64,65]. However, to our knowledge, significant attraction of *P. mimicus* to racemic *anti*-2,3-octanediol but not to the individual (2*R*,3*S*)- and (2*S*,3*R*)-enantiomers, may be the first documented case of enantiomeric synergism within the subfamily Cerambycinae.

The recent development of hierarchical modeling frameworks has greatly expanded the potential for analyzing data on species communities. The analyses presented here represent a novel application of Hierarchical Modeling of Species Communities (HMSC; [39]) and we were able to obtain a far more informative output than would have been possible with previous approaches, such as ordination. Selecting the 26 species with ten or more individuals, we were able to produce informative models for 18 species (as defined by a predictive *r*^2^ of >0.1). However, neither the incidence nor abundance models performed well for eight species. This may reflect insufficient data (notably seven of the eight species had <50 individuals across 44 trapping locations) or it may suggest that the factors we examined were not important in determining their distribution. Such species could be habitat generalists or, conversely, their distributions may be determined by small-scale factors, such as the presence of particular host tree species, that were not captured in our plot scale analysis.

Our models demonstrated that trap height, elevation, slope, and LAI were all important factors determining the incidence and abundance of species. In the incidence model (Figure 2a), there was support for a positive association with LAI for seven species. At low elevation, most species (11 out of 13) were more likely to occur in high LAI plots, whereas this trend diminished at higher elevation, generating a negative elevation:LAI interaction. Among most species (10 out of 13) there was support for a positive association with slope, although for *Megopis costipennis* the opposite was true. This may reflect forest quality rather than slope per se, because the steepest slopes occurred in less accessible valleys with largely undisturbed forest. Four species (*M. costipennis*, *D. pseudonotabilis, R. laosica*, *A. lineata*) showed support for a positive trap height:elevation interaction, indicating an increased tendency to occur in the canopy traps at higher elevations. Five species (*M. costipennis*, *D. pseudonotabilis, X. lateralis fracturis*, *X. incurvatus*, *D. theresae*) also tended to occur at higher elevation. The abundance (condition-on-presence) model further informed our understanding of the effects of environmental factors (Figure 2b). Several species showed strong support for increased captures in the canopy traps (7 out of 11 species), at higher elevations (4 out of 11 species), and on steeper slopes (7 out of 11 species), but reduced captures in plots with higher LAI (8 out of 11 species). However, three species showed support for a negative association with elevation and two with slope. Variable responses among species indicate that the community is segregating according to these environmental factors. Elevation and slope were important determinants of community composition among the tree species [24] and hence these differences may reflect the host preferences. Moreover, it might be expected that species that utilize living trees would be found in the canopy, whereas species that infest long dead, fallen trees would be found closer to ground level. Nonetheless, our results were analogous to several similar recent studies with attractant-baited traps, which found that whereas a subset of the total number of cerambycid species caught might be trapped preferentially in high or low traps, few or no species were caught exclusively at one height e.g., [10,27,28,29,66].

Interestingly, many of the responses seen across species were also captured when looking at the collective responses at the subfamily level for Cerambycinae (Figure 2c,d) but not for Lamiinae, suggesting that these environmental responses may be driven by traits conserved among Cerambycinae. There was only one species-to-species association with significant support, between *M*. *costipennis* and *R*. *horsfieldi* in the abundance model. This is interesting because *M. costipennis* was attracted to (2*R*,3*S*)-2,3-octanediol [19] and *R. horsfieldi* was significantly attracted to (2*S*,3*R*)-2,3-octanediol (vs. controls) in this study. Perhaps the species-to-species association in response to the generic lures reflects common attraction to the environment around traps or it may reflect pheromone signaling before they were captured. *M. costipennis* produces (2*R*,3*S*)-2,3-octanediol [19], while *R*. *horsfieldi* likely produces a multi-component pheromone, of which (2*R*,3*S*)-2,3-octanediol and (2*S*,3*R*)-2,3-octanediol may be components (Table 2), though captures to *syn*-2,3-hexanediols and *syn*-2,3-octanediols were higher than *anti*-2,3-octanediols (see Figure 2 in [13]). Thus, the species-species association is likely attributed to their presence in the same environment coupled by the attraction of each species to their own pheromone components, and no inhibition of attraction of *M*. *costipennis* to racemic *anti*-2,3-octanediol [19]. Regardless of the overlap of pheromone components and communication channels, cross attraction is completely avoided because *M*. *costipennis* is nocturnal and *R*. *horsfieldi* is diurnal (JW, pers. obs.) Finally, looking at the overall community responses, it is clear that the diversity of species is higher in canopy traps and on steeper slopes, but there is a decline in species richness with elevation and LAI (Figure 3).

In summary, the results reported here provide a number of new leads for identification of the pheromones of species which were caught in substantial numbers during the course of this study, including some species with the potential to be invasive. This study also demonstrated the potential value of pheromone-based trapping as a tool for assessment of the cerambycid fauna of a site or region. Such assessments are becoming increasingly important, both in the context of monitoring endangered species and in the context of monitoring for incursions of invasive species. Finally, the HMSC analyses allowed a finer-grained assessment of the effects of site and trapping variables on the responses of beetles, providing insights into their ecology.

## Figures and Tables

**Table 1:** Total numbers of each of the 71 species of cerambycids caught at 22 field sites in traps baited with multicomponent pheromone lures, and the code used for species names. At each site, 3 traps were deployed ~1 m above ground, and another 3 traps were hung at ~18 m high in the canopy. Species caught in numbers of 10 or more for which pheromones or likely pheromonal attractants have been identified previously are shown in bold font.

Species	Total #	Code
Cerambycinae		
*Artimpaza lineata* (Pic)	168	Arti lin
*Ceresium nilgiriense* Gahan	1	Cere nil
*Ceresium sinicum* White	1	Cere sin
*Chlorophorus arciferus* (Chevrolat)	3	Chlo arc
*Chlorophorus reductus* Pic	7	Chlo red
*Chlorophorus rubricollis* (Castelnau and Gory)	2	Chlo rub
*Demonax alcanor* Gressitt and Rondon	1	Demo alc
***Demonax gracilestriatus*** Gressitt and Rondon	230	Demo gra
*Demonax occultus* Gressitt and Rondon	24	Demo occ
*Demonax pseudonotabilis* Gressitt	262	Demo pse
***Demonax theresae*** Pic	43	Demo the
*Euryphagus lundii* F.	1	Eury lun
*Gnatholea eburifera* Thomson	2	Gnat ebu
*Gnatholea subnuda* Lacordaire	4	Gnat sub
*Perissus atronotatus* Pic	10	Peri atr
*Perissus dilatus* Gressitt and Rondon	6	Peri dil
*Perissus griseus* Gressitt	47	Peri gri
*Perissus mimicus* Gressitt and Rondon	333	Peri mim
*Perissus mutabilis obscuricolor* Pic	14	Peri mut
*Rhaphuma anongi* Gressitt and Rondon	113	Rhap ano
*Rhaphuma circumscripta* (Schwarzer)	2	Rhap cir
***Rhaphuma horsfieldi*** (White)	468	Rhap hor
***Rhaphuma laosica*** Gressitt and Rondon	201	Rhap lao
*Rhaphuma patkaina* Gahan	1	Rhap pat
*Xoanodera maculata* Schwarzer	10	Xoan mac
***Xylotrechus buqueti*** (Castelnau and Gory)	23	Xylo buq
***Xylotrechus chinensis*** (Chevrolat)	11	Xylo chi
*Xylotrechus diversignatus magdelainei* Pic	3	Xylo div
***Xylotrechus incurvatus*** (Chevrolat)	66	Xylo inc
*Xylotrechus lateralis* Gahan	3	Xylo latf
*Xylotrechus lateralis fracturis* Guo and Chen	75	Xylo lat
*Xylotrechus unicarinatus* Pic	2	Xylo uni
*Xylotrechus wauthieri* Gressitt and Rondon	3	Xylo wau
Lamiinae		
*Acalolepta basiplagiata* (Breuning)	2	Acal bas
***Acalolepta formosana*** (Breuning)	28	Acal for
*Alidus biplagiatus* Gahan	1	Agel ton
*Agelasta tonkinea* Pic	1	Alid bip
*Arctolamia fasciata* Gestro	1	Arct fas
*Blepephaeus fulvus* (Pic)	1	Blep ful
*Blepephaeus stigmosus* Gahan	1	Blep sti
*Blepephaeus succinctor* (Chevrolat)	1	Blep suc
*Cacia yunnana* Breuning	5	Caci yun
*Coptops annulipes* Gahan	1	Copt ann
*Coptops leucostictica rustica* Gressitt	3	Copt leu
*Diastocera wallichi* (Hope)	12	Dias wal
*Euseboides matsudai* Gressitt	1	Euse mat
*Glenea diverselineata intermedia* Breuning	12	Glen div
*Glenea relicta formosensis* Breuning	1	Glen rel
*Imantocera penicillata* (Hope)	2	Iman pen
*Mesocacia multimaculata* (Pic)	1	Meso mul
*Mesosa rupta* (Pascoe)	5	Meso rup
*Mispila khamvengae* Breuning	1	Misp kha
*Mispila sonthianae* Breuning	2	Misp son
***Monochamus bimaculatus*** Gahan	78	Mono bim
*Monochamus dubius* Gahan	14	Mono dub
*Olenecamptus siamensis* Breuning	1	Olen sia
*Paraleprodera carolina* Fairmaire	1	Para car
*Paraleprodera diophthalma* (Pascoe)	1	Para dio
*Paraleprodera stephanus fasciata* Breuning	1	Para ste
***Pharsalia subgemmata*** (Thomson)	1601	Phar sub
*Pseudocalamobius discolineatus* Pic	2	Pseu dis
***Pseudomacrochenus antennatus*** (Gahan)	78	Pseu ant
*Pseudonemorphas versteegi* (Ritsema)	1	Pseu ver
*Pterolophia lateralis* Gahan	1	Pter lat
*Pterolophia serricornis* Gressitt	4	Pter ser
*Pterolophia subtubericollis* Breuning	3	Pter sub
*Sthenias gracilicornis* Gressitt	9	Sthe gra
*Uraecha punctata* Gahan	3	Urae pun
***Xenohammus bimaculatus*** Schwarzer	32	Xeno bim
Prioninae		
*Dorysthenes huegelii* Redtenbacher	2	Dory hue
***Megopis costipennis*** White	487	Mego cos
Total	4541	

**Table 2:** Numbers (mean ± SE) of beetles caught in traps baited with (2*R*,3*S*)-2,3-octanediol (RS-diol), (2*S*,3*R*)-2,3-octanediol (SR-diol), racemic *anti*-2,3-octanediol (racemate), or a solvent control (*R* and *S* denote the configuration of the chiral center). Different letters (a, b) indicate significant different in mean numbers of beetles captured.

	Treatment				Friedman’s Q (df)	
Species	RS-diol	SR-diol	Racemate	Control		p
**Cerambycinae**						
Tribe Clytini						
*Perissus mimicus*	0 ^b^	0 ^b^	2.8 ± 0.85 ^a^	0 ^b^	14.6 (3,16)	0.0022
*Rhaphuma horsfieldi*	1.6 ± 0.1 ^ab^	2.5 ± 0.76 ^a^	1.8 ± 0.5 ^ab^	0 ^b^	12.9 (3,32)	0.0048

**Table 3:** Model performance for the Hierarchical Modelling of Species Communities (HMSC). We used the predictive *r*^2^, which was based on a five-fold cross validation, to determine model performance. Species with values in italics were not well predicted in either model. Note that *Pharsalia subgemmata* saturated the traps such that an incidence model could not be estimated.

Species	Incidence Model (Probit) Turj *r*^2^		Abundance Model (Lognormal Poisson) *r*^2^	
	Statistical	Predictive	Statistical	Predictive
*Pharsalia subgemmata*	NA	NA	0.672	0.297
*Megopis costipennis*	0.364	0.224	0.932	0.281
*Rhaphuma horsfieldi*	0.114	0.024	0.801	0.365
*Perissus mimicus*	0.461	0.163	0.823	0.612
*Demonax pseudonotabilis*	0.387	0.132	0.226	−0.048
*Demonax gracilestriatus*	0.365	0.044	0.775	0.303
*Rhaphuma laosica*	0.375	0.278	0.518	0.142
*Artimpaza lineata*	0.381	0.271	0.529	0.097
*Rhaphuma anongi*	0.532	0.411	0.672	0.429
*Pseudomacrochenus antennatus*	0.218	0.061	0.604	0.253
*Monochamus bimaculatus*	*0.228*	*0.026*	*0.364*	*0.019*
*Xylotrechus lateralis fracturis*	*0.439*	0.142	*0.593*	−*0.011*
*Xylotrechus incurvatus*	0.313	0.253	0.635	0.367
*Perissus griseus*	*0.243*	*0.063*	*0.213*	−*0.018*
*Demonax theresae*	0.246	0.146	0.474	*0.097*
*Xenohammus bimaculatus*	0.298	0.175	0.528	0.169
*Acalolepta formosana*	*0.145*	*0.005*	*0.051*	−*0.095*
*Xylotrechus buqueti*	0.504	0.399	0.100	*0.000*
*Demonax occultus*	*0.178*	*0.039*	*0.243*	*0.000*
*Monochamus dubius*	0.237	0.104	0.032	−0.357
*Perissus mutabilis obscuricolor*	*0.127*	*0.022*	*0.459*	−*0.029*
*Xoanodera maculata*	0.223	−0.115	0.893	0.321
*Diastocera wallichi*	*0.191*	*0.065*	*0.643*	−*0.002*
*Glenea diverselineata intermedia*	*0.118*	*-0.063*	*0.432*	−*0.229*
*Xylotrechus chinensis*	*0.215*	*0.037*	*0.381*	−*0.115*
*Perissus atronotatus*	0.463	0.300	0.571	−0.063

## Data Availability

See Supplementary Materials.

## Supplementary Materials

The following are available online at https://www.mdpi.com/2075-4450/12/4/277/s1, HMSC Graphical Output, R-code, and Raw Data. Figure S1. AlphaScents Black Panel Trap, Table S1. Plot Characteristics Table, Table S2. Comparison of 2010 vs. 2013 studies.
